# Short-Term Precipitation Radar Echo Extrapolation Method Based on the MS-DD3D-RSTN Network and STLoss Function

**DOI:** 10.3390/s24155004

**Published:** 2024-08-02

**Authors:** Wulin Yang, Hao Yang, Hang Zhou, Yuanchang Dong, Chenghong Zhang, Chaoping Chen

**Affiliations:** 1 School of Computer Science, Chengdu University of Information Technology, Chengdu 610225, China; 3220608028@stu.cuit.edu.cn (W.Y.); zhouhang@cuit.edu.cn (H.Z.); 2 Heavy Rain and Drought-Flood Disaster in Plateau and Basin Key Laboratory of Sichuan Province, Institute of Plateau Meteorology, China Meteorological Administration (CMA), Chengdu 610072, China; dongychang@163.com (Y.D.); ipmzhang@163.com (C.Z.); 3 Sichuan Meteorological Observatory, Chengdu 610071, China; chaopingchen2023@163.com

**Keywords:** short-term precipitation forecasting, radar echo extrapolation, spatiotemporal convolution, loss function, deep learning

## Abstract

Short-term precipitation forecasting is essential for agriculture, transportation, urban management, and tourism. The radar echo extrapolation method is widely used in precipitation forecasting. To address issues like forecast degradation, insufficient capture of spatiotemporal dependencies, and low accuracy in radar echo extrapolation, we propose a new model: MS-DD3D-RSTN. This model employs spatiotemporal convolutional blocks (STCBs) as spatiotemporal feature extractors and uses the spatial-temporal loss (STLoss) function to learn intra-frame and inter-frame changes for end-to-end training, thereby capturing the spatiotemporal dependencies in radar echo signals. Experiments on the Sichuan dataset and the HKO-7 dataset show that the proposed model outperforms advanced models in terms of CSI and POD evaluation metrics. For 2 h forecasts with 20 dBZ and 30 dBZ reflectivity thresholds, the CSI metrics reached 0.538, 0.386, 0.485, and 0.198, respectively, representing the best levels among existing methods. The experiments demonstrate that the MS-DD3D-RSTN model enhances the ability to capture spatiotemporal dependencies, mitigates forecast degradation, and further improves radar echo prediction performance.

## 1. Introduction

Short-term precipitation forecasting is a key component of modern meteorological forecasting systems, focusing on accurately predicting drastic changes in precipitation caused by severe convective weather within the next six hours, particularly within the 0–2 h range [1,2,3]. In recent years, severe weather disasters due to heavy precipitation have caused substantial social and economic losses. For example, in 2021, Zhengzhou, China, experienced an extreme precipitation event with a maximum hourly rainfall of 201.9 mm, resulting in 380 deaths and missing persons, and direct economic losses amounting to CNY 40.9 billion [4]. Thus, short-term precipitation forecasting is critically important in sectors like agriculture, transportation, urban management, and tourism. It is essential for disaster prevention and protecting lives and property [5,6,7]. Enhancing the accuracy of short-term precipitation forecasts and providing timely predictions of rainfall are crucial research needs.

Meteorological radar is one of the core meteorological detection devices. By emitting microwave signals and receiving their reflections, it can effectively detect various atmospheric elements and be used for precipitation forecasting [8]. China has established a new generation weather radar network, which can obtain high-resolution radar echo data with minute-level temporal resolution and kilometer-level spatial resolution over large areas [9]. These devices and data provide feasibility for research on short-term precipitation forecasting. Developing precise short-term precipitation forecasting methods using radar echo data has become one of the current research hotspots and bottlenecks. Radar echo extrapolation-based short-term precipitation forecasting methods need to capture subtle atmospheric changes within a short time, facing significant challenges in accuracy, real-time performance, and technical requirements. The core concept of the radar echo extrapolation method is to predict future frames of radar echo images based on past frames, thereby forecasting future precipitation. This requires developing spatiotemporal sequence prediction models and obtaining the optimal solution [10]. However, modeling the spatiotemporal sequence characteristics using radar data is challenging due to the high-dimensional nonlinearity and extremely complex spatiotemporal distribution of the data, making it difficult to develop accurate spatiotemporal sequence prediction methods.

Traditional radar echo extrapolation methods often rely on mathematical and physical approaches, such as the cross-correlation [11], storm cell identification and tracking (SCIT) [12], and optical flow methods [13]. Although these methods can predict precipitation distribution to some extent, their ability to capture spatiotemporal relationships is limited, especially when dealing with the nonlinear motion of mesoscale atmospheric processes. Therefore, traditional methods cannot accurately forecast changes in precipitation. In recent years, with significant improvements in computing power, radar echo extrapolation methods based on deep learning [14] have shown better performance than traditional methods. Deep learning-based radar echo extrapolation methods capture spatiotemporal features and nonlinearity from large amounts of radar echo data to extrapolate future frames of radar echo images. Compared to traditional methods, using deep learning for radar echo extrapolation has advantages in data utilization and forecasting accuracy [15,16,17,18,19,20]. Currently, recurrent neural networks (RNN) and convolutional neural networks (CNN) are the mainstream methods for handling spatiotemporal sequence prediction tasks in radar echo extrapolation. RNN-based models are primarily designed for modeling spatiotemporal sequence data. They have strong capabilities for temporal sequence modeling and can incrementally learn and predict radar echo sequences. However, they also have limitations, such as the inability to perform parallel computation, gradient explosion, and the accumulation of errors. To address these issues, researchers have proposed using CNN architecture networks for radar echo extrapolation, such as U-Net [21] and SimVP [22] models. U-Net is a groundbreaking convolutional neural network architecture that consists of two symmetrical modules: an encoder and a decoder. It not only excels in semantic segmentation [23], visual detection [24], and medical tasks [25], but has also been extensively studied and proven to be an effective backbone model in the field of precipitation forecasting. SimVP, a new architecture model featuring a CNN-CNN-CNN structure, adds a translator module between the encoder and decoder of U-Net to learn temporal evolution, which enhances its ability to capture temporal information compared to the U-Net network. However, 2D convolution operations mix originally independent variables into indistinguishable feature channels, losing the independence of features and making it difficult to reflect the interdependencies between variables. This approach does not fully capture the temporal dependencies of radar echo data, still exhibiting significant prediction degradation, insufficient capture of temporal dependencies at different time scales, and low accuracy.

To address existing issues, we propose an end-to-end short-term precipitation forecasting model based on radar echo extrapolation: Multi-Scale Deep Dilated 3D Residual Spatio-Temporal Network (MS-DD3D-RSTN). This model uses multi-scale deep and dilated 3D convolutions to extract spatiotemporal features while significantly reducing the issue of excessive parameter quantities. The introduction of residual connections helps alleviate prediction degradation. A new loss function, STLoss, combines weighted mean squared error (WMSE) and differential divergence regularization (DDR) to learn intra-frame and inter-frame changes in radar data, effectively capturing the spatiotemporal variation trends of radar signals. The combination of these innovative designs enables the STCB module to more effectively capture correlated features in both temporal and spatial dimensions, thereby improving the model’s performance in radar echo extrapolation tasks. To evaluate the effectiveness of this method, we conducted experiments on the Sichuan dataset and the HKO-7 dataset. The results show that the proposed method achieves superior performance in terms of the CSI and POD evaluation metrics. Specifically, the CSI metrics reached 0.538 and 0.386 for the 20 dBZ reflectivity threshold and 0.485 and 0.198 for the 30 dBZ reflectivity threshold, demonstrating superior performance compared to existing radar extrapolation methods.

The main contributions of this work are as follows:

(1) A spatiotemporal sequence learning network model, MS-DD3D-RSTN, is proposed, which efficiently captures the spatiotemporal dependencies of radar echo data and accurately predicts the target task.
(2) The STCB module, based on multi-scale 3D convolution, dilated deep convolution, and residual connections, is proposed to achieve better spatiotemporal dependency capture capabilities and alleviate prediction degradation to some extent.
(3) We introduced a loss function, STLoss, which combines WMSE and DDR. This effectively addresses data imbalance issues and enhances the model’s ability to learn spatiotemporal features and their gradient characteristics.

The remainder of the paper is organized as follows: Section 2 briefly introduces related work on radar echo extrapolation. Section 3 describes the proposed method. Section 4 presents comprehensive experiments to validate the effectiveness of the proposed model. Section 5 summarizes the innovations of this paper and discusses the advantages and disadvantages of related methods. Section 6 concludes the paper.

## 2. Related Work

In recent years, deep learning techniques have made significant advancements in various fields such as computer vision, image recognition, natural language processing, and spatiotemporal prediction. Deep learning methods effectively train models on large datasets to learn the nonlinear relationships within the data [26,27,28,29]. Consequently, deep learning-based radar echo extrapolation has also garnered considerable attention from researchers [30,31]. Below, we introduce deterministic models based on RNN and CNN for radar echo extrapolation.

### 2.1. RNN-Based Radar Echo Extrapolation Models

In 2015, Shi et al. [10] proposed the ConvLSTM network model based on FC-LSTM to address the spatiotemporal sequence problem in precipitation forecasting. This method effectively captures spatiotemporal correlations, opening a new perspective for precipitation forecasting. They later realized that natural movements and transformations are generally location-variant, indicating a flaw in the location-invariant convolutional recurrent structure of the ConvLSTM model. In 2017, Shi et al. [32] proposed the TrajGRU model, which can actively learn the location-variant structure of recurrent connections, improving the accuracy of precipitation nowcasting. They also established a benchmark including a large-scale dataset, a new training loss function, and comprehensive evaluation protocol to advance research and establish evaluation standards in this field. In 2017, Wang et al. [33] introduced the PredRNN model, recognizing that both spatial appearance and temporal changes are crucial for spatiotemporal sequence prediction. They advocated for simultaneously memorizing spatial appearances and temporal changes in a unified memory pool for more effective predictions. To this end, they designed a novel LSTM unit with a zigzag motion structure to extract and memorize key spatial and temporal features simultaneously. The following year, they introduced the PredRNN++ model [34], which incorporates a new recurrent structure, cascaded dual memory, and a gradient highway unit to adaptively capture long- and short-term temporal dependencies while mitigating gradient propagation issues in deep predictive models. In 2019, Kun et al. [35] proposed the E3D-LSTM model, which combines 3D convolution with RNN to enhance the network’s local motion perception capability. The model utilizes a gated self-preserving module to achieve a long-term memory “inheritance” call, effectively learning short-term frame dependencies and long-term high-level relationships. In 2020, Guen et al. [36] introduced a physics-based model, PhyDNet, which separates partial differential equation (PDE) dynamics and supplementary information through a dual-branch deep architecture. The model integrates recurrent physical units (PhyCell) and ConvLSTM units to learn physical dynamics and residual information, showing advantages in disentangling and long-term prediction. In 2021, Chang et al. [37] proposed a motion-aware unit (MAU) that expands the temporal receptive field of prediction units through attention and fusion modules, effectively capturing inter-frame motion information and retaining visual details through an information recall scheme. In 2022, Wang et al. [38] further improved PredRNN by decoupling memory units and introducing a zigzag memory flow to facilitate the exchange of visual dynamics at different levels. They also employed a memory decoupling loss and a curriculum learning strategy to optimize feature learning and long-term dynamic capture.

### 2.2. CNN-Based Radar Echo Extrapolation Models

Agrawal et al. [3] were the first to use the original U-Net model for precipitation prediction in the continental United States, verifying its performance. Song et al. [39] combined U-Net, ResNet, squeeze-and-excitation, and spatial attention modules to construct the SE-ResUNet model, predicting rainfall dynamics in Beijing for the following two hours. Kevin et al. [40] proposed an improved U-Net model, SmaAt-UNet, which combines attention mechanisms and depthwise separable convolutions, demonstrating a significant reduction in trainable parameters while maintaining prediction performance. Broad-UNet [41] features asymmetric parallel convolutions and a spatial pyramid pooling module, which learns more complex patterns by combining multi-scale features to predict precipitation within the Netherlands. Shen et al. [42] proposed an ADC_Net model based on dilated convolution and attention convolution for radar echo extrapolation. This model retains the internal data structure of the feature matrix, extracts multi-scale spatial features, and utilizes attention convolution to enhance sensitivity to target features and suppress interference, effectively improving radar echo extrapolation accuracy. SimVP [22] is a simple video prediction model built entirely on CNNs. Daehyeon et al. [43] applied SimVP to precipitation forecasting, achieving excellent performance across various precipitation conditions in South Korea over a 120 min forecast period. NowcastNet [44] is a data-driven nonlinear forecasting model that combines physical evolution schemes and conditional learning. It generates physically plausible high-resolution, long-term, and multi-scale detailed extreme precipitation forecasts over large areas through end-to-end error optimization, effectively addressing extreme precipitation events associated with advection or convection processes.

## 3. Methodology

### 3.1. Datasets

This study uses the Sichuan dataset and the HKO-7 dataset as experimental datasets, with the same model being trained separately on each. Through analysis of the raw data, we discovered a significant number of negative values. Generally, higher radar echo reflectivity indicates greater precipitation; therefore, these negative values can be ignored. First, all negative values in the raw data were set to zero, and noise filtering was applied to the dataset. The data were normalized to facilitate model training and optimization. To ensure the quality of the training samples, we excluded most samples without precipitation. After screening and preprocessing the raw data, we generated a sample dataset using a sliding window with a length of 40 and a step size of 1.

Sichuan dataset: This dataset is derived from radar echo data collected by the Plateau Meteorological Bureau of Sichuan Province, China, from 2011 to 2013. The data are three-dimensional, comprising nine layers. Images have a resolution of 360 × 920 pixels, spanning from 105.09° E to 109.95° E in longitude and from 29.09° N to 33.25° N in latitude. Based on experimental results, radar echoes from the first, third, and fifth layers were selected for analysis. The corresponding altitudes are 0.5 km, 1.5 km, and 2.5 km, respectively. After processing, the dataset consists of 10,394 samples, each with a sequence length of 40 frames at 6 min intervals. The first 20 frames of the first, third, and fifth layers are used as input for prediction, while the subsequent 20 frames of the first layer serve as the ground truth.

HKO-7 dataset: This dataset, developed by the Hong Kong Observatory, is commonly used for precipitation nowcasting. It includes radar echo data collected from 2009 to 2015. The images have a resolution of 480 × 480 pixels, with an altitude of 2 km. Centered on Hong Kong, the coverage area is 512 km × 512 km. After processing, the dataset contains 11,514 samples, with each sample sequence having a length of 40 frames and a time interval of 6 min [32].

Both datasets are divided into training, validation, and test sets in a 7:2:1 ratio.

### 3.2. Problem Definition

In the field of radar echo extrapolation for near-term precipitation forecasting, the spatiotemporal sequence prediction problem can be modeled as a prediction problem based on historical radar image sequences [10]. Specifically, the data flow of this problem can be represented as follows: Given a specific time point *t*, the data of the *D* time points prior to *t* are used as historical input data, with a time interval of 6 min between data points. Let $T_{1}=\left\{t-D,t-D+1,\cdots ,t\right\}$ be the sequence of data points within *D* time points before *t*, which are input into the prediction model to forecast radar images for the *M* time points $T_{2}=\left\{t+1,t+2,\cdots ,t+M\right\}$ after *t*. The prediction interval is also 6 min, and the input data for each time point is a three-dimensional matrix $I_{i}$ of size C×H×W.

For the historical input data, it can be represented as a dataset $I=\{I_{t-D},I_{t-D+1},\cdots ,I_{t}\}$, with dimensions D×C×H×W. For the predicted radar image sequence at future time points, it is represented as $P=\{P_{t+1},P_{t+2},\cdots ,P_{M}\}$, with dimensions D×1×H×W.

To address this problem, the MS-DD3D-RSTN model, as an objective function *F*, can be used to construct a mathematical model that maps the historical input dataset I to the predicted future radar image sequence *P*. The specific mathematical expression is as follows:

(1) $$F\left(I=\left\{I_{t-D},I_{t-D+1},\cdots ,I_{t}\right\}\right)=\left\{P_{t+1},P_{t+2},\cdots ,P_{M}\right\}.$$

### 3.3. MS-DD3D-RSTN Network Framework

**Figure 1:**
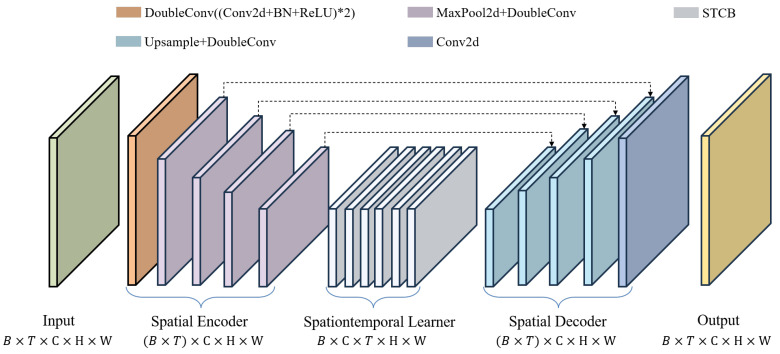
Model framework.

Figure 1 illustrates the network framework of the MS-DD3D-RSTN model. The model consists of three parts: the spatial encoder, the spatiotemporal learner, and the spatial decoder. The spatial encoder and spatial decoder are symmetric modules. The spatial encoder learns spatial information, reduces spatial dimensions, and decreases the number of parameters; the spatial decoder is responsible for mapping the feature information to the target sequence to predict the target task. The spatiotemporal learner learns spatiotemporal evolution and captures the temporal dependencies of radar echo data. To retain spatially related features, multiple skip connections are added between the spatial encoder and the spatial decoder. The input size of the model is (20,3,H,W), and the output size is (20,1,H,W), indicating that the model uses radar reflectivity maps from three layers, two hours before, as input to predict a single layer of radar images for the next two hours.

The core function of the spatial encoder lies in extracting spatial feature information and performing dimensionality reduction, with a primary focus on the spatial dimension. To achieve this, the radar echo image tensor of past frames (B×T×C×H×W) is first converted into a tensor of shape ((B×T)×C×H×W). The converted tensor data is then processed using DoubleConv (DoubleConv is a dual convolutional layer, where each convolutional layer comprises a 3×3 Conv2D, a batch normalization (BN) layer, and the activation function ReLU) (represented by the brown blocks in Figure 1) to increase the hidden dimensions, facilitating subsequent operations. The next step involves stacking $N_{e}$ modules of MaxPool2d (The stride is set to 2, and the kernel size is 2×2) and DoubleConv combinations (represented by the light purple blocks in Figure 1) to downsample and extract spatial features. The hidden features in the spatial encoder can be represented as

(2) $$z_{i}=\mathrm{DoubleConv}\left(x\right),i=0$$

(3) $$z_{i}=\mathrm{MaxPool}2d\left(\mathrm{DoubleConv}\left(z_{i-1}\right)\right),1\leq i\leq N_{e}$$

where the dimensions of the input tensor *x* and the output tensor $z_{i}$ are ((B×T)×C×H×W) and $\left(\left(B\times T\right)\times C^{\prime }\times H^{\prime }\times W^{\prime }\right)$, respectively. DoubleConv represents a combination of two layers of Conv2d, BatchNormal, and ReLu, and $N_{e}$ is the number of MaxPool2d and DoubleConv combination modules. Experiments have shown that the optimal size for $N_{e}$ is 4.

The spatiotemporal learner primarily focuses on the temporal dimension. Therefore, the output tensor of the encoder ((B×T)×C×H×W) is converted into a tensor of shape (B×C×T×H×W) to arrange the same variables sequentially along the time dimension. Then, by stacking $N_{t}$ STCB modules (represented by the light gray blocks in Figure 1), temporal features are extracted from the converted tensor. The STCB module is specifically introduced in Section 3.3. The hidden features in the spatiotemporal learner can be represented as

(4) $$z_{j}=\mathrm{STCB}\left(z_{j-1}\right),N_{e}<j\leq N_{e}+N_{t}$$

where the dimensions of the input tensor $z_{j-1}$ and the output tensor $z_{j}$ are (B×C×T×H×W) and $\left(B\times C^{\prime }\times T\times H\times W\right)$, respectively. $N_{t}$ is the number of STCB modules. Experiments have shown that the optimal size for $N_{t}$ is 6.

The primary function of the spatial decoder is to integrate feature information and predict the radar reflectivity images of future frames. Corresponding to the spatial encoder, the output tensor of the spatiotemporal learner (B×T×C×H×W) is first converted into a tensor of shape ((B×T)×C×H×W). Then, by stacking $N_{d}$ modules of Upsample (the scaling factor is set to 2, and bilinear interpolation is used for upsampling) and DoubleConv combinations (represented by the light blue blocks in Figure 1), feature information is integrated from the feature tensors of the spatial encoder and spatiotemporal learner. Finally, a 1×1 convolutional layer is used to output the predicted images. The hidden features in the spatial decoder can be represented as

(5) $$z_{k}=\mathrm{DoubleConv}\left(\mathrm{Upsample}\left(z_{k-1}\right)\right),N_{e}+N_{t}<k\leq N_{e}+N_{t}+N_{d}$$

(6) $$z_{k}=\mathrm{Conv}2d\left(z_{k-1}\right),k=N_{e}+N_{t}+N_{d}+1$$

where the dimensions of the input tensor $z_{k-1}$ and the output tensor $z_{k}$ are ((B×T)×C×H×W) and $\left(\left(B\times T\right)\times C^{\prime }\times H^{\prime }\times W^{\prime }\right)$, respectively. $N_{d}$ is the number of Upsample and DoubleConv combination modules. The specific value of $N_{d}$ is the same as $N_{e}$.

### 3.4. STCB

**Figure 2:**
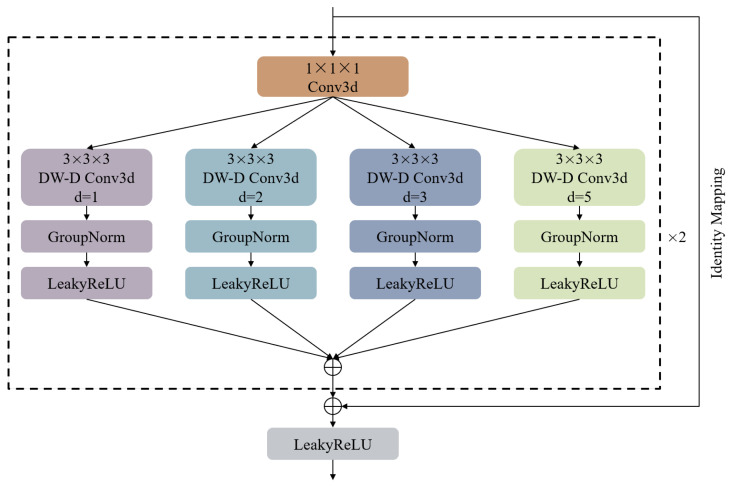
Structure of the STCB.

The STCB module, as the core component of the temporal learner, delves deeply into the dynamic features of sequential data. It integrates network techniques such as multi-scale 3D convolution, dilated depthwise convolution (DW-D), and residual connections, forming a network module designed to precisely capture subtle temporal motion changes, as shown in Figure 2.

The specific design of the STCB module is as follows: In the first step, a 1×1×1 3D convolution is used at the very beginning to increase the hidden dimension for subsequent operations. In the second step, a multi-branch architecture is implemented using 3×3×3 dilated depthwise 3D convolutions (DW-D Conv3d) layers with different dilation rates (d = 1, 2, 3, 5). The output tensor from the second step is fed into four branches, each containing a 3×3×3 DW-D Conv3d layer followed by a GroupNorm normalization layer and a LeakyReLU activation function. In the third step, the different feature information extracted by each branch in the second step is integrated. In the fourth step, the above operations are repeated. In the fifth step, residual connections are applied by performing element-wise addition between the initial input tensor and the output tensor from the fourth step. In the sixth step, the output tensor from the fourth step is passed through a LeakyReLU activation function to obtain the final result of the STCB module.

Given the multidimensional time series nature of radar reflectivity images, traditional 2D convolution operations might mix originally independent variables, leading to a loss of independence among feature channels and making it difficult to reflect the interrelationships between variables. Therefore, this study employs 3D convolution technology to explore the interdependencies across temporal and spatial scales, thus more accurately capturing the spatiotemporal features of radar reflectivity images.

The distribution of key information in spatiotemporal data is complex and dynamically changing, so the model needs to handle information at different scales flexibly. This study achieves multi-scale feature extraction by applying convolution kernels of different sizes. Smaller convolution kernels are used to capture fine local features, while larger convolution kernels are used to capture globally distributed information. Additionally, the multi-branch architecture design allows the model to effectively integrate local details and global trends.

However, large-sized 3D convolution kernels may lead to reduced computational efficiency and a significant increase in model parameters [45]. To address this issue, this study employs dilated depthwise convolution to achieve different receptive field sizes while reducing the number of model parameters. Specifically, the STCB module introduces residual connections to retain original feature information, which alleviates prediction degradation to some extent and improves the model’s ability to capture long-term dependencies. Although the STCB module is built on a purely convolutional network, it can effectively capture spatiotemporal dependencies.

### 3.5. Loss Functions

We introduce a novel loss function, STLoss, which consists of two components: WMSE [39] and DDR [46]. These components are used to learn intra-frame and inter-frame changes in radar data, respectively. The introduction of WMSE addresses the issue of precipitation sample imbalance. By setting thresholds, this component helps adjust the model’s emphasis on precipitation regions of varying intensities, ensuring that the model can balance the influence of different precipitation intensities during prediction. On the other hand, to overcome the shortcomings of the MSE loss that only considers intra-frame errors, DDR is proposed to learn the temporal variation trends of the data. It helps understand the differences between consecutive frames and captures the inherent changes in the data. The design of this combined loss function not only effectively handles data imbalance but also promotes better learning of spatiotemporal features by the model, thereby further improving the model’s accuracy.

The specific implementation of STLoss is as follows:

(1) Calculate the intra-frame error
  We calculate the intra-frame error between the real and predicted radar echo images using a weighted mean squared error. Different weights $w_{i}$ are assigned based on the various ranges of radar reflectivity. The mean squared error is then calculated between the predicted values $y_{i}^{\prime }$ and the target values $y_{i}$. Finally, the errors for different ranges are multiplied by their corresponding weights $w_{i}$. The specific formula is as follows:
  (7) $$WMSE=\frac{1}{t}\sum_{i=1}^{t}w_{i}\cdot {\left(y_{i}^{\prime }-y_{i}\right)}^{2},$$
  where *t* is the prediction length, and the weights $w_{i}$ are defined as: $w_{i}=\left\{\begin{array}{cc} 1, & y_{i}<20\\ 2, & 20\leq y_{i}<30\\ 4, & 30\leq y_{i}<40\\ 6, & 40\leq y_{i} \end{array}\right.$.
  Data analysis revealed that the occurrence frequency of different precipitation intensities is highly imbalanced. Based on the data distribution in various intervals, the weights $w_{i}$ are set to 1, 2, 4, and 6, respectively, to amplify the prediction errors for different radar reflectivity intervals by the corresponding multiples.
(2) Calculate the inter-frame error
  We calculate the inter-frame error between the real and predicted radar echo images using differential sparsity regularization. First, the differences between adjacent frames in the time dimension for both the predicted values $y^{\prime }$ and the target values *y* need to be computed. The specific formula is as follows:
  (8) $$\begin{array}{cc} \Delta y_{i}^{\prime } & =y_{i+1}^{\prime }-y_{i}^{\prime }\\ \Delta y_{i} & =y_{i+1}-y_{i} \end{array}.$$
  Next, the difference matrix is flattened into a one-dimensional vector. The softmax function [47,48,49,50] is then applied to convert the differences into a probability distribution. The specific formula is as follows:
  (9) $$\begin{array}{cc} \sigma (\Delta y^{\prime }) & =softmax(\Delta y^{\prime })\\ \sigma (\Delta y) & =softmax(\Delta y) \end{array}.$$
  Finally, the Kullback–Leibler (KL) divergence method [51] is used to measure the difference between the two probability distributions. The specific formula is as follows:
  (10) $$\begin{array}{cc} DDR & =D_{KL}\left(\sigma \left(\Delta Y^{\prime }\right)∥\sigma \left(\Delta Y\right)\right)\\ & =\sum_{i=1}^{t-1}\left(\Delta Y_{i}^{\prime }\right)\log\frac{\sigma \left(\Delta Y_{i}^{\prime }\right)}{\sigma \left(\Delta Y_{i}\right)}, \end{array}$$
  where $D_{KL}$ denotes the KL divergence method, σ(ΔY) is the probability distribution of the target values, $\sigma (\Delta Y^{\prime })$ is the probability distribution of the predicted values, and *t* is the prediction length.
(3) Calculate the target loss function, STLoss
  The STLoss consists of two parts: the weighted mean squared error and the discrepancy disentangled regularization, with α and β representing the corresponding constant weights. The specific formula is as follows:
  (11) STLoss=α·WMSE+β·DDR.

### 3.6. Implementation Details

This experiment is conducted under the PyTorch framework, using ADAM as the optimizer to train the model. The batch size is set to 2, and the learning rate during training is set to $1\times 10^{-3}$. The MS-DD3D-RSTN model uses STLoss as the loss function. The GPU used in the experiment is the GeForce RTX 4090 with 24 GB of memory. The specific experimental parameter settings are shown in Table 1.

### 3.7. Evaluation Metrics

We use a threshold-based evaluation method to test the test set, with thresholds selected at 20, 30, and 40 dBZ. The prediction durations include 1 h and 2 h, with a forecast interval of 6 min. The evaluation metrics adopted are the commonly used meteorological indicators: Critical Success Index (CSI), Probability of Detection (POD), and False Alarm Ratio (FAR) [52,53,54]. CSI is a comprehensive scoring standard used to evaluate the accuracy of quantitative precipitation forecasts. POD refers to the proportion of correctly identified actual precipitation areas in the forecast. FAR measures the proportion of incorrectly predicted precipitation areas out of all predicted precipitation areas. Therefore, the higher the values of POD and CSI, and the lower the value of FAR, the more accurate the prediction results, and the better the model performance. The specific formulas are as follows:

(12) $$CSI=\frac{\mathrm{hit}}{\mathrm{hit}+\mathrm{miss}+\mathrm{far}}$$

(13) $$POD=\frac{\mathrm{hit}}{\mathrm{hit}+\mathrm{miss}}$$

(14) $$FAR=\frac{\mathrm{far}}{\mathrm{hit}+\mathrm{far}},$$

where hit represents true positives, meaning both the predicted and actual values are above the threshold; miss represents false negatives, where the predicted value is below the threshold, but the actual value is above it; and far represents false positives, where the predicted value is above the threshold, but the actual value is below it.

## 4. Experimental Results and Analysis

### 4.1. Results Comparison

**Figure 3:**
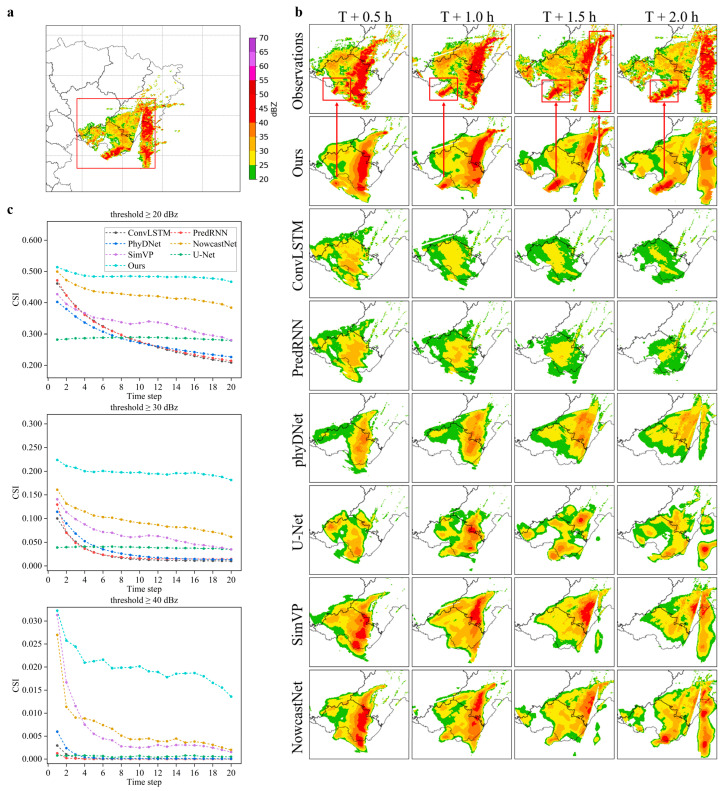
Comparison experiment visualization results: (**a**) overall forecast area; (**b**) time series forecast results of the red boxed area in (**a**) compared with the forecast results of other models; (**c**) comparison of forecast indicators at different time steps within 2 h.

To validate the effectiveness of the proposed method, the MS-DD3D-RSTN model was compared with six baseline models on the Sichuan dataset using different reflectivity thresholds in extrapolation experiments. Six advanced benchmark models in the task of radar echo extrapolation were selected: ConvLSTM [10], PredRNN [33], PhyDNet [36], U-Net [21], SimVP [22], and NowcastNet [44]. The experiments were conducted for a 2 h extrapolation period (20 time steps), evaluating the average results at reflectivity thresholds of 20 dBz, 30 dBz, and 40 dBz as shown in Table 2, and for a 1 h extrapolation (10 frames) and a 2 h extrapolation (20 frames) as shown in Table 3. Additionally, to visually observe the data evolution, evaluation metric plots for different prediction steps are presented in Figure 3c. The analysis of the results reveals the following insights:

(1) Using CSI, POD, and FAR as binary classification metrics to assess the accuracy of predicting heavy precipitation, the MS-DD3D-RSTN model demonstrates superior performance in terms of CSI and POD across all reflectivity thresholds compared to existing methods. Specifically, CSI values reach 0.485, 0.198, and 0.020 at 20 dBZ, 30 dBZ, and 40 dBZ reflectivity thresholds, respectively. However, its performance on FAR metrics needs improvement, indicating a need to reduce false alarms.
(2) As the reflectivity intensity increases and the forecast duration extends, all models show a significant decline in forecasting ability. Notably, the MS-DD3D-RSTN model exhibits a slower decline compared to other models, particularly excelling in tasks involving strong radar echoes. Its performance in handling long-term dependencies is remarkable, effectively capturing complex spatiotemporal relationships among data, thereby enhancing accuracy and stability in long-term prediction tasks.

In Figure 3b, visual results of a case analysis are provided to further validate the model’s predictive capabilities:

(1) Extrapolation images from the MS-DD3D-RSTN model demonstrate closer resemblance to real images in terms of shape and spatial changes. Moreover, these images retain more strong echo areas in the later extrapolation periods, mitigating prediction degradation issues.
(2) Specific local regions marked with red boxes and arrows in Figure 3 illustrate significant changes in echo intensity. The MS-DD3D-RSTN model accurately predicts the trend of radar echo sequences, closely matching actual conditions in most regions. Overall, the MS-DD3D-RSTN model not only enhances the accuracy of short-term precipitation forecasting to some extent but also exhibits commendable performance in handling long-term dependencies.

### 4.2. Ablation Study

**Figure 4:**
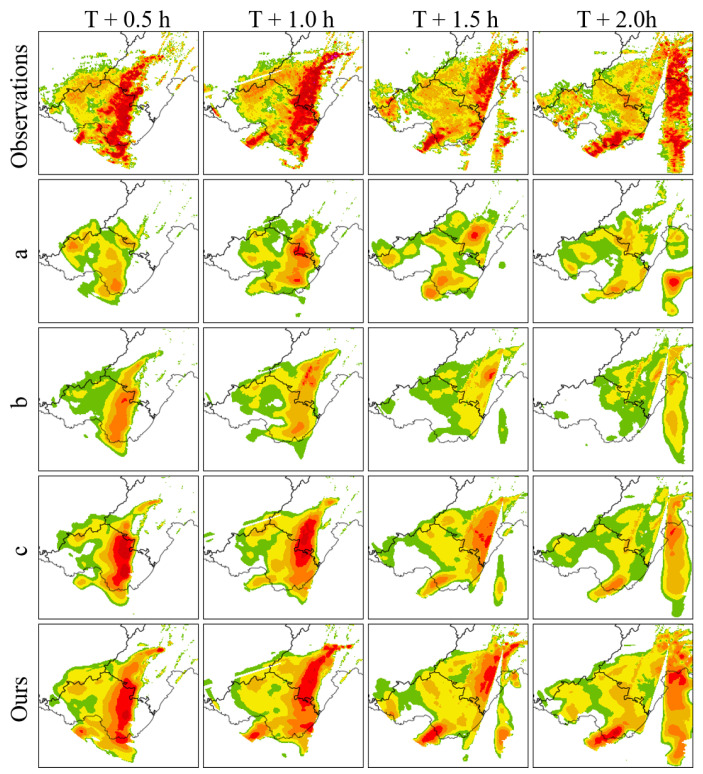
Comparison of visualization results of ablation study: a represents excluding STCB and STLoss; b represents residual connections and STLoss excluding STCB; c represents excluding STLoss.

To evaluate the contribution and effect of each component in the MS-DD3D-RSTN model, ablation experiments were conducted on the STCB module, residual connections within the STCB module, and the STLoss function using the Sichuan dataset. From Table 4 and Table 5 and Figure 4, the following conclusions can be drawn:

(1) Removing the STCB module results in the CSI indices differing by 19.9%, 15.9%, and 1.9% under 20 dBZ, 30 dBZ, and 40 dBZ reflectivity thresholds, respectively. Additionally, in visual images, the model with the STCB module predicts the intensity and area of the echoes more accurately than the model without the STCB module. Therefore, it can be seen that the STCB module enhances the ability to capture spatiotemporal dependencies to a certain extent.
(2) Removing the residual connections within the STCB module results in the CSI indices differing by 13.0%, 14.6%, and 1.8% under 20 dBZ, 30 dBZ, and 40 dBZ reflectivity thresholds, respectively. Additionally, in visual images, the inclusion of residual connections in the STCB module leads to more accurate predictions of echo intensity, especially in the later stages. Therefore, it can be seen that the STCB module alleviates the problem of model prediction degradation to a certain extent.
(3) Removing the STLoss function results in the CSI indices differing by 7.3%, 10.6%, and 1.4% under 20 dBZ, 30 dBZ, and 40 dBZ reflectivity thresholds, respectively. Additionally, in visual images, using the STLoss function results in more accurate predictions of the area and intensity of echoes at medium and high thresholds. Therefore, it can be seen that the STLoss function improves the prediction accuracy of medium and strong echoes to a certain extent.

### 4.3. Robustness Study

To verify the robustness of the model, we conducted robustness experiments on the HKO-7 dataset, comparing the MS-DD3D-RSTN model with the better-performing models from Section 4.2. Table 6 shows the quantitative data of the final forecast results of meteorological indicators under different radar reflectivity thresholds for the SimVP, NowcastNet, and MS-DD3D-RSTN models on the test set. The results clearly indicate that the MS-DD3D-RSTN model performs best in terms of CSI and POD metrics, with CSI scores reaching 0.538, 0.386, and 0.126 at the 20 dBZ, 30 dBZ, and 40 dBZ reflectivity thresholds, respectively, demonstrating its superior ability to accurately predict the true radar distribution compared to other models.

**Figure 5:**
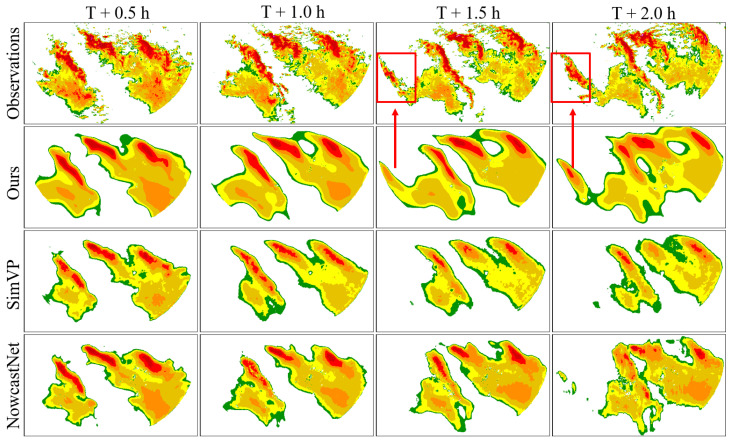
Comparison of visualization results of robustness study.

To further illustrate the predictive performance of the model, we selected typical examples from the test dataset and compared the visual results of different models at 0.5 h, 1.0 h, 1.5 h, and 2.0 h, as shown in Figure 5. As indicated by the red boxes and red arrows in the local regions of Figure 5, the MS-DD3D-RSTN model can more accurately predict the appearance distribution of radar echoes, with a trajectory that matches the actual path, retaining more information. Compared to other models, it alleviates prediction degradation to a certain extent and improves prediction accuracy.

In summary, the MS-DD3D-RSTN model exhibits superior performance on different datasets compared to other models, maintaining stable and reliable capabilities.

## 5. Discussion

We propose an end-to-end radar echo extrapolation-based nowcasting model: MS-DD3D-RSTN. The main contributions include the introduction of a spatiotemporal learner and the STLoss function. The spatiotemporal learner, composed of stacked STCB modules, focuses on dynamic changes in time and space, capturing the spatiotemporal dependencies of radar echoes. The STCB is a multi-branch architecture that utilizes multi-scale depth and dilated 3D convolutions to perform convolutions at different temporal and spatial scales. It cleverly employs residual connections to mitigate prediction degradation to some extent. Additionally, the STLoss function is introduced to learn changes within and between radar frames, enhancing the model’s ability to learn the dynamic changes of radar echoes at different temporal and spatial scales, further improving the model’s accuracy, as confirmed by the prediction results.

The core of radar echo extrapolation methods based on recurrent neural networks lies in the recurrent units, which achieve continuous memory and updating of temporal information through recurrent connections, capturing dynamic features in the data. However, recurrent structures also have some significant drawbacks. Firstly, due to the presence of recurrent structures, errors accumulate during the prediction process, especially with long time series, potentially leading to decreased prediction accuracy. Secondly, the characteristics of recurrent structures make parallel processing inefficient, reducing computational efficiency, particularly on large datasets. In tasks such as radar echo extrapolation prediction, these issues result in prediction degradation and low accuracy. The proposed method in this paper, however, strives to preserve all detailed information to prevent prediction degradation and improve model prediction accuracy.

Radar echo extrapolation methods based on convolutional neural networks essentially adopt an encoder-decoder network architecture. This architecture, through symmetric contraction and expansion paths, demonstrates strong feature extraction capabilities, effectively focusing on the texture features of radar echoes, and accelerates the prediction process through parallel computation. However, radar echoes exhibit dynamic variability at different temporal and spatial scales. Conventional convolution operations are performed only on the spatial scale, neglecting temporal dependencies, and thus cannot capture spatiotemporal correlations well, leading to poor performance in radar echo extrapolation tasks. To overcome these issues, the proposed method employs multi-scale 3D convolutions to focus on temporal and spatial dimensions. Additionally, the STLoss function emphasizes the information gradient differences between sequential frames, forcing the network to focus on the temporal evolution of radar signals, thereby more efficiently capturing the features of radar temporal extrapolation.

The radar echo extrapolation model MS-DD3D-RSTN proposed in this paper is a deep learning model purely based on radar data analysis. However, the precipitation process is influenced by various factors such as atmospheric physics and meteorology. Although the MS-DD3D-RSTN model has shown improvements in certain areas, there are still some shortcomings. Firstly, the model has many parameters, requiring substantial computational resources. Secondly, as the prediction time extends, both the accuracy of the predictions and the clarity of the images tend to decrease. Future research can focus on physics-based deep learning methods to enhance the model’s physical interpretation of precipitation generation and evolution, further improving the performance of radar echo extrapolation.

The MS-DD3D-RSTN model has broad potential applications in practice. Firstly, it can be used to improve meteorological forecasting systems, especially in predicting heavy rainfall events, thereby enhancing disaster prevention and mitigation efficiency. Secondly, the model can be deployed in the field of agricultural management, providing farmers with more accurate weather forecasts to help optimize planting and harvesting schedules. Additionally, accurate precipitation forecasts are crucial in urban management for preventing urban flooding and planning infrastructure. Given these application scenarios, further improving the accuracy of the model’s predictions is of significant practical importance.

## 6. Conclusions

We propose a novel radar echo extrapolation model, MS-DD3D-RSTN, which uses a spatiotemporal learner with stacked STCB modules to capture spatiotemporal dependencies and employs the STLoss function for end-to-end training. Comparative experiments with state-of-the-art models on the test dataset demonstrate its superior performance in the task of radar echo extrapolation for nowcasting precipitation. Future work will explore incorporating the physical laws underlying the precipitation process to further improve the model’s prediction accuracy.

## Figures and Tables

**Table 1:** Parameter Settings.

Parameter	Value
Historical Sequence Length	20
Prediction Sequence Length	20
Optimizer	ADAM
Loss Function	MSE, STLoss
Batch Size	2
Learning Rate	0.001

**Table 2:** Results of 1 h Extrapolation Test in Comparative Experiment. The values in bold are the top-1 results.

Model	r≥20 dBZ			r≥30 dBZ			r≥40 dBZ		
	CSI ↑	POD ↑	FAR ↓	CSI ↑	POD ↑	FAR ↓	CSI ↑	POD ↑	FAR ↓
ConvLSTM	0.346	0.398	0.280	0.038	0.040	0.327	0.000	0.000	**0.012**
PredRNN	0.347	0.397	0.269	0.040	0.043	0.333	0.000	0.000	0.015
PhyDNet	0.323	0.373	0.294	0.050	0.057	0.389	0.001	0.001	0.030
U-Net	0.287	0.350	0.353	0.040	0.048	0.375	0.001	0.001	0.013
SimVP	0.362	0.415	0.268	0.085	0.095	0.362	0.009	0.010	0.109
NowcastNet	0.445	0.511	**0.230**	0.112	0.123	**0.297**	0.009	0.010	0.072
Ours	**0.490**	**0.653**	0.345	**0.203**	**0.255**	0.435	**0.023**	**0.027**	0.101

**Table 3:** Results of 2 h Extrapolation Test in Comparative Experiment. The values in bold are the top-1 results.

Model	r≥20 dBZ			r≥30 dBZ			r≥40 dBZ		
	CSI ↑	POD ↑	FAR ↓	CSI ↑	POD ↑	FAR ↓	CSI ↑	POD ↑	FAR ↓
ConvLSTM	0.291	0.331	0.287	0.025	0.026	0.319	0.000	0.000	**0.006**
PredRNN	0.293	0.332	0.280	0.028	0.029	0.336	0.000	0.000	0.009
PhyDNet	0.284	0.326	0.306	0.033	0.037	0.406	0.001	0.001	0.017
U-Net	0.286	0.351	0.356	0.039	0.046	0.383	0.001	0.001	0.014
SimVP	0.337	0.386	0.275	0.067	0.075	0.368	0.006	0.007	0.086
NowcastNet	0.426	0.490	**0.237**	0.095	0.103	**0.307**	0.006	0.006	0.052
Ours	**0.485**	**0.647**	0.349	**0.198**	**0.250**	0.458	**0.020**	**0.024**	0.096

**Table 4:** Results of 1 h Extrapolation Test in Ablation Study. a represents the model without STCB and STLoss; b represents the model without residual connections in STCB and STLoss; c represents the model without STLoss. The values in bold are the top-1 results.

Model	r≥20 dBZ			r≥30 dBZ			r≥40 dBZ		
	CSI ↑	POD ↑	FAR ↓	CSI ↑	POD ↑	FAR ↓	CSI ↑	POD ↑	FAR ↓
a	0.287	0.350	0.353	0.040	0.048	**0.375**	0.001	0.001	**0.013**
b	0.368	0.442	0.294	0.059	0.067	0.407	0.002	0.003	0.051
c	0.422	0.506	**0.288**	0.101	0.116	0.389	0.007	0.008	0.065
Ours	**0.490**	**0.653**	0.345	**0.203**	**0.255**	0.453	**0.023**	**0.027**	0.101

**Table 5:** Results of 2 h Extrapolation Test in Ablation Study. a represents the model without STCB and STLoss; b represents the model without residual connections in STCB and STLoss; c represents the model without STLoss. The values in bold are the top-1 results.

Model	r≥20 dBZ			r≥30 dBZ			r≥40 dBZ		
	CSI ↑	POD ↑	FAR ↓	CSI ↑	POD ↑	FAR ↓	CSI ↑	POD ↑	FAR ↓
a	0.286	0.351	0.356	0.039	0.046	**0.383**	0.001	0.001	**0.014**
b	0.355	0.424	0.298	0.052	0.060	0.413	0.002	0.002	0.048
c	0.412	0.495	**0.291**	0.092	0.105	0.392	0.006	0.006	0.055
Ours	**0.485**	**0.647**	0.349	**0.198**	**0.250**	0.458	**0.020**	**0.024**	0.096

**Table 6:** Results of 2 h Extrapolation Test in Robustness Study. The values in bold are the top-1 results.

Model	r≥20 dBZ			r≥30 dBZ			r≥40 dBZ		
	CSI ↑	POD ↑	FAR ↓	CSI ↑	POD ↑	FAR ↓	CSI ↑	POD ↑	FAR ↓
SimVP	0.430	0.479	**0.209**	0.220	0.250	**0.300**	0.045	0.050	**0.229**
NowcastNet	0.497	0.564	0.214	0.320	0.386	0.330	0.086	0.098	0.352
Ours	**0.538**	**0.694**	0.310	**0.386**	**0.577**	0.447	**0.126**	**0.160**	0.397

## Data Availability

The HKO-7 dataset can be found at https://github.com/sxjscience/HKO-7/blob/master/README.md. Restrictions apply to the availability of the Sichuan dataset. The dataset was obtained from the Plateau Meteorological Bureau of Sichuan Province, China, and is available from the authors with the permission of a third party.
